# The human dimension of fire regimes on Earth

**DOI:** 10.1111/j.1365-2699.2011.02595.x

**Published:** 2011-12

**Authors:** David M J S Bowman, Jennifer Balch, Paulo Artaxo, William J Bond, Mark A Cochrane, Carla M D'Antonio, Ruth DeFries, Fay H Johnston, Jon E Keeley, Meg A Krawchuk, Christian A Kull, Michelle Mack, Max A Moritz, Stephen Pyne, Christopher I Roos, Andrew C Scott, Navjot S Sodhi, Thomas W Swetnam, Robert Whittaker

**Affiliations:** 1 School of Plant Science, Private Bag 55, University of TasmaniaHobart, Tas., Australia; 2 NCEAS735 State Street, Suite 300University of Santa BarbaraSanta Barbara, CA, USA; 3 Instituto de Física, Universidade de São Paulo 1516Rua do Matão, Travessa R, 187, São Paulo, SP, Brazil; 4 Botany Department, University of Cape TownRondebosch, South Africa; 5 Geographic Information Science Center of Excellence (GIScCE) South Dakota State UniversityBrookings, SD, USA; 6 Environmental Studies Program and Department of Ecology, Evolution and Marine Biology, University of CaliforniaSanta Barbara, CA, USA; 7 Ecology, Evolution & Environmental Biology, Columbia UniversityNew York, NY, USA; 8 Menzies Research Institute, University of TasmaniaPrivate Bag 23, Hobart, Tas., Australia; 9 US Geological Survey, Western Ecological Research Center, Sequoia-Kings Canyon Field StationThree Rivers, CA, USA; 10 Department of Ecology and Evolutionary Biology, University of CaliforniaLos Angeles, CA, USA; 11 Department of Environmental Science, Policy and Management, University of CaliforniaBerkeley, CA, USA; 12 School of Geography and Environmental Science, Monash UniversityMelbourne, Vic., Australia; 13 Department of Biology, University of FloridaGainesville, FL, USA; 14 Environmental Science, Policy, and Management Department, University of CaliforniaBerkeley, CA, USA; 15 School of Life Sciences, Arizona State UniversityTempe, AZ, USA; 16 Department of Anthropology, Southern Methodist UniversityDallas, TX, USA; 17 Department of Earth Sciences, Royal Holloway University of LondonEgham, UK; 18 Department of Biological Sciences, Faculty of Science, National University of SingaporeSingapore; 19 Laboratory of Tree-Ring Research, The University of ArizonaTucson, AZ, USA

**Keywords:** Fire and culture, fire management, fire regime, global environmental change, landscape fire, palaeoecology, prehistoric human impacts, pyrogeography

## Abstract

Humans and their ancestors are unique in being a fire-making species, but ‘natural’ (i.e. independent of humans) fires have an ancient, geological history on Earth. Natural fires have influenced biological evolution and global biogeochemical cycles, making fire integral to the functioning of some biomes. Globally, debate rages about the impact on ecosystems of prehistoric human-set fires, with views ranging from catastrophic to negligible. Understanding of the diversity of human fire regimes on Earth in the past, present and future remains rudimentary. It remains uncertain how humans have caused a departure from ‘natural’ background levels that vary with climate change. Available evidence shows that modern humans can increase or decrease background levels of natural fire activity by clearing forests, promoting grazing, dispersing plants, altering ignition patterns and actively suppressing fires, thereby causing substantial ecosystem changes and loss of biodiversity. Some of these contemporary fire regimes cause substantial economic disruptions owing to the destruction of infrastructure, degradation of ecosystem services, loss of life, and smoke-related health effects. These episodic disasters help frame negative public attitudes towards landscape fires, despite the need for burning to sustain some ecosystems. Greenhouse gas-induced warming and changes in the hydrological cycle may increase the occurrence of large, severe fires, with potentially significant feedbacks to the Earth system. Improved understanding of human fire regimes demands: (1) better data on past and current human influences on fire regimes to enable global comparative analyses, (2) a greater understanding of different cultural traditions of landscape burning and their positive and negative social, economic and ecological effects, and (3) more realistic representations of anthropogenic fire in global vegetation and climate change models. We provide an historical framework to promote understanding of the development and diversification of fire regimes, covering the pre-human period, human domestication of fire, and the subsequent transition from subsistence agriculture to industrial economies. All of these phases still occur on Earth, providing opportunities for comparative research.

## Introduction

Debate over the concept of ‘background’ natural processes (i.e. independent of humans) and over the role of humans in driving global environmental change requires an understanding of the influence of anthropogenic fire on ecological systems (Crutzen, 2002; Crutzen & Steffen, 2003; ACIA, 2004; Steffen *et al.*, 2007; Zalasiewicz *et al.*, 2011). What recommends fire as an ideal topic through which to investigate human–environmental coupling is its leading role in an ancient narrative about how humans and the Earth have interacted (Goudsblom, 1992). Humans enjoy a monopoly over fire's use; indeed, its possession is a defining trait of humanity. We have made fire a near-universal catalyst for most of our exchanges with the world around us, from technology to land use. Our continuous use of fire is culturally framed and transmitted, and it continues to undergo rapid changes in expression (Pyne, 2000). Oddly, given the significance of fire to humanity and to the Earth system, there is little understanding of this interplay. Appreciation of the evolving relationships and geographic patterns of anthropogenic landscape burning are crucial because the survival of many species and ecosystems hinges on understanding the historical range of variability in fire activity (Jackson & Hobbs, 2009; Keane *et al.*, 2009). This perspective also has substantial implications for the intellectual basis of fire management that extends beyond the sciences into the realm of cultural values, illuminating how different cultures think about fire and how their institutions seek to manage it (Goudsblom, 1992; Pyne, 2000, 2001; Laris, 2002). Our purpose here is to describe the historical development of humanity's relationship with fire and the role humans play in the diversification of contemporary fire regimes globally, thus providing a framework for thinking about how humans influence fire regimes in space and time.

## Prehistoric human influence on fire regimes

Charcoal in the sedimentary record reveals continuous fire activity on Earth since the late Silurian, a period of roughly 400 million years (Scott, 2000, 2010). Fire has been an important selective factor for plant evolution and has shaped the development of some biomes (Simon *et al.*, 2009; Crisp *et al.*, 2010, 2011), for example contributing to the diversification and spread of angiosperms (Bond & Scott, 2010) and to the development and global spread of highly flammable savannas in the late Cenozoic (Keeley & Rundel, 2005), the habitat in which human ancestors evolved. The ubiquity of background natural fire activity in these environments makes it impossible to date when hominins began to use fire, although archaeological deposits provide reliable evidence for routine controlled use of fire by the Middle Pleistocene, 690–790 ka (Goren-Inbar *et al.*, 2004; Roebroeks & Villa, 2011). It has been suggested that fire may have contributed to the evolution of *Homo* species by enabling the cooking of food as early as the Lower Pleistocene (Wrangham, 2009).

**Figure 1:**
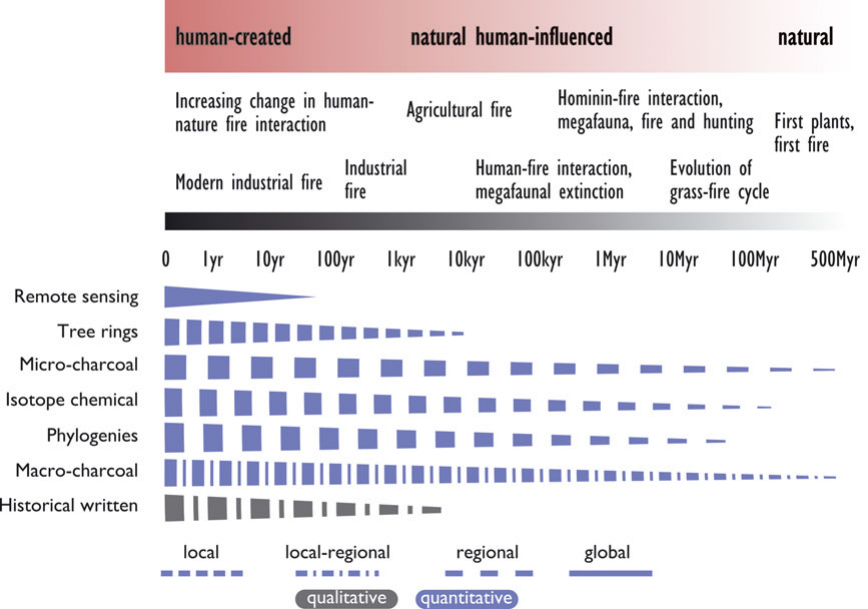
Summary of the available historical sources and palaeoecological proxies to reconstruct fire regimes, spanning the period from the advent of fire on Earth in deep time to the modern industrial period characterized by fossil fuel combustion. The spatial and temporal resolution of all these approaches varies and decays with increasing time depth, constraining our understanding of fire regimes, especially before the Industrial Revolution.

Reconstructions of palaeofire regimes for different ecosystems and biomes rely on a diverse range of proxies, with variable spatial and temporal resolutions (Whitlock *et al.*, 2010) (Fig. 1). These data generate debate, particularly about the overall importance of anthropogenic burning in the distant past. Nonetheless, virtually all palaeofire scientists, biogeographers and anthropologists recognize that humans have used fire over sustained periods of time for a plethora of local-scale, domestic purposes and to modify nearby habitats. Disagreements arise over the spatial extent of human modification, whether these effects were intentional or unintentional, and the degree to which humans overrode otherwise natural fire regimes. These disagreements are especially vigorous for regions and time periods with relatively low human population densities and/or high lightning strike densities that provide plentiful natural ignition sources, such as tropical savannas. The primary means by which early humans influenced fire regimes was by increasing the number of ignitions and changing their timing, as well as by altering fuel structure and abundance. The impact of increased numbers of human-caused fires is relative to background rates of natural ignitions. In environments more or less saturated by lightning ignitions, humans would have contributed relatively little to the frequency of ignitions, although they could have substantially altered the seasonal timing and locations of ignitions and altered fuels by introducing grazing animals or hunting megafauna (Savage & Swetnam, 1990; Flannery, 1994; Gill *et al.*, 2009).

**Figure 2:**
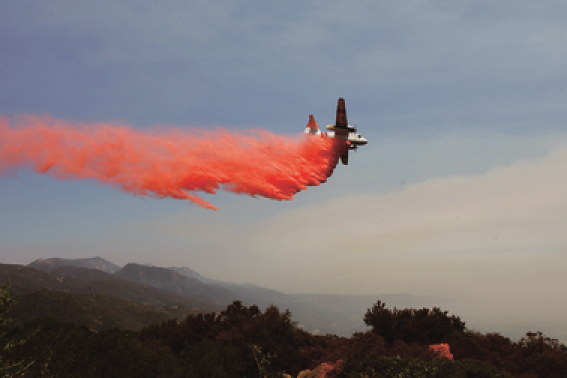
Sophisticated fire-fighting technologies, such as aerial drops of fire retardants, have been developed to control fire activity in flammable landscapes, especially where humans have established settlement or valuable infrastructure. With permission from David McNew, Getty Images.

Three important sets of information are needed to distinguish human burning reliably in the palaeoecological record: (1) temporal or spatial changes in fire activity and vegetation apparent from palaeoecological proxies (see Fig. 1), (2) a demonstration that these changes are not predicted by climate–fuels–fire relationships and palaeoclimate reconstructions for the period of fire regime change, and (3) a demonstration that fire regime changes coincide in space and time with changes in human history (e.g. technological, economic, political, or demographic changes, including colonization of new lands) known from archaeology, anthropology and historical sources. For these reasons, oceanic islands that were infrequently burnt prior to human colonization in the late Holocene, such as New Zealand, with its high-resolution and well-dated sedimentary records, are ideal systems through which to study the ecological impact of anthropogenic burning. For example, McWethy *et al.* (2010) show that the colonization of the south island of New Zealand 800 years ago was marked by a rapid burst of burning and associated loss of forest cover, which failed to recover in lower-rainfall regions. Here, the flora had few species with adaptations to recover from fire (Bond *et al.*, 2004), and charcoal in the sedimentary record suggested that fire occurred less than twice per millennium prior to human settlement. It is much harder to detect the impact of humans in Australia, given that fire has been an important feature of the environment since at least the late Cenozoic and the flora has developed adaptations to survive and recover from burning (Crisp *et al.*, 2010, 2011; He *et al.*, 2011). Furthermore, human colonization occurred in the late Pleistocene, at the limit of radiocarbon dating, so archaeological sites are few and difficult to date reliably. Thus, determining, for example, if fire was a cause or an effect of the extinction of the Australian megafauna in the late Pleistocene remains controversial given the available data. Miller *et al.* (2005) suggest that sustained Aboriginal burning triggered ‘ecosystem collapse’ by degrading habitats, in contrast to the suggestion that over-hunting of large herbivores caused increased fuel build-up and thus subsequent changes to fire regimes (Flannery, 1994). Alternative plausible views are that: (1) overhunting, not fire, was pivotal to the extinctions (Roberts & Brook, 2010), (2) there was an interaction among effects of human impacts (Brook & Bowman, 2002), or (3) there was possibly a human–climate interaction (Murphy *et al.*, 2011).

The separation of the anthropogenic effect from ‘natural’ background fire regimes remains a source of debate in both academic and management communities. To many people, the concept of ‘natural’ fire regimes can only be applied to regions in the period prior to human colonization and settlement. But this use is problematic for fire regimes in Africa, where humans evolved. Confusingly, the term is sometimes used to describe all prehistoric fire regimes, whether or not they were dependent on human or lightning ignitions. For example, the USA Landscape Fire and Resource Management Planning Tools (LANDFIRE) includes Native American burning patterns within natural fire regimes. In the USA and Canada, forest fires ignited by lightning that do not threaten human populations or infrastructure are sometimes allowed to burn freely to allow the maintenance of ‘natural’ disturbances in these ecosystems. The intertwined relationships between humans, landscapes and fire throughout human history argue against a clear distinction between natural and anthropogenic fires. The notion of ‘restoring natural fire regimes’ without anthropogenic influence is neither possible nor useful. However, understanding the relative influences of climate, human ignition sources, and cultural practices in particular settings underpins efforts to minimize damage from fire to human health, property, and ecosystems, and to limit greenhouse gas fluxes to the atmosphere.

## Contemporary human fire regimes

Fire regimes can be thought of as a spatially variable template of fire intensity, severity, type, frequency, spatial scale and seasonality, within which biotas have co-evolved (Gill, 1975; Pausas & Keeley, 2009). Humans influence fire regimes in a multitude of ways, including by changing fuel types, modifying fuel structure and continuity, and igniting few or many fires in different seasons under various weather conditions (Tables 1 & 2). Motivations for manipulating fire regimes vary considerably and include arson and warfare, skilful management of natural resources (e.g. agriculture, ranching, forestry and wildlife management) and protection of infrastructure and urban areas. Despite the diversity and sophistication of fire use, humans cannot completely control the fires they set, nor always limit the spread of fires caused by natural ignitions. Some uncontrolled fires can be destructive, causing economic disruption, loss of life, damage to physical and mental health, and degradation of natural resources (e.g. pollution of air and water, losses of biodiversity and soil) (Cameron *et al.*, 2009; Johnston, 2009). Nevertheless, fire is crucial for the functioning of many ecosystems, and thus in the provision of ecological services and the maintenance of biological diversity. Many human cultures, therefore, have an ambiguous relationship with landscape fires, which can create political tensions amongst groups with competing models of fire management. A contemporary global example concerns the deliberate use of fire to clear tropical rain forests – a process that can generate tension internationally if the smoke crosses national borders (Lohman *et al.*, 2007). At a local scale, air pollution in urban areas can be a side effect of deliberately set fires prescribed to decrease the risk of severe wildfires in flammable landscapes (Bowman & Johnston, 2005; Kochi *et al.*, 2010).

**Table 1:** How humans influence fire regime parameters by modifying key variables that affect fire activity

Fire variable	Natural influences	Human influences	Fire regime parameters
Wind speed	Season Weather Topography Land cover	Climate change Land cover change	Fire spread
Fuel continuity	Terrain type (slope, rockiness, aspect) Rivers and water bodies Season Vegetation (type, age, phenology)	Artificial barriers (roads, fuel breaks) Habitat fragmentation (fields) Exotic grasses Land management (patch burning, fuel treatments) Fire suppression	
Fuel loads	Tree, shrub and grass cover Natural disturbances (e.g. insect or frost damage, windthrow) Herbivory Soil fertility Season	Grazing Timber harvests Exotic species establishment Fire suppression Fuel treatments Land use and land cover (deforestation, agriculture, plantations)	Fire intensity and severity
Fuel moisture	Season Antecedent precipitation Relative humidity Air temperature Soil moisture	Climate change Land management (logging, grazing, patch burning) Vegetation type and structure (species composition, cover, stem density)	
Ignition	Lightning Volcanoes Season	Human population size Land management Road networks Arson Time of day Season Weather conditions	Number and spatial and temporal patterns of fires

**Table 2:** Examples of how fire regimes have changed during the industrial era, from a representative cross-section of biomes from low to high latitudes. This ongoing transition is described in Fig. 3, in which pre-industrial fire regimes correspond to pyric phases C and D, and post-industrial fire regimes correspond to pyric phases E and F

Biome	Pre-industrial fire regime	Post-industrial fire regime
Tropical rain forest	Very infrequent low-intensity surface fires with negligible long-term effects on biodiversity	Frequent surface fires associated with forest clearance causing a switch to flammable grassland or agricultural fields
Tropical savanna	Frequent fires in dry season causing spatial heterogeneity in tree density	Reduced fire due to heavy grazing causing increased woody species recruitment
Mid-latitude desert	Infrequent fires following wet periods that enable fuel build-up	Frequent fires due to the introduction of alien flammable grasses
Mid-latitude North American seasonally dry forests	Frequent low-intensity surface fires limiting recruitment of trees	Fire suppression causing high densities of juveniles and infrequent high-intensity crown fires
Boreal forest	Infrequent high-intensity crown fires causing replacement of entire forest stands	Increased high-intensity wildfires associated with global warming causing loss of soil carbon and switch to treeless vegetation

Although there is a general relationship between human population density and fire activity in some regions, these variables are not necessarily linearly related, being strongly influenced by the environmental setting (Venevsky *et al.*, 2002; Syphard *et al.*, 2007; Li *et al.*, 2009). Small populations of humans can significantly increase the ignition density if these populations are highly mobile or inhabit landscapes where background fire activity is low, such as on islands where the target area for lightning is small (Wardle *et al.*, 1997). Indeed, some of the most notorious examples of ecological impacts of anthropogenic burning come from islands where background fire activity was low (Olson & James, 1982; McWethy *et al.*, 2010). The effect of humans on background fire regimes also varies markedly across gradients in primary productivity (McWethy *et al.*, 2010; Whitlock *et al.*, 2010), as outlined below.

### Fire in high primary productivity environments

Tropical rain forests rarely burn naturally because of the very low coincidence of lightning with climate conditions suitable to carry fire. Even if a fire is initiated, the high moisture content in fuels generally prevents propagation. However, tropical rain forests can be completely transformed by human-set fires. Small-scale agriculturalists typically burn very small areas of tropical forests using slash and burn methods, but during exceptionally hot and dry years these fires may unintentionally spread over much wider areas. Fires set during droughts are also used to clear tropical rain forest to establish broad-scale agriculture, and fire spread in adjacent forests is further facilitated by the selective logging that typically precedes the agriculture frontier, creating a powerful feedback cycle (Cochrane, 2003). Furthermore, previously burnt rain forests are more likely to burn again. For example, fires during 1997–2006 burnt about 21% of forest cover on the tropical forested island of Borneo, with 6% of the land being burnt more than once (Langner & Siegert, 2009). Anthropogenic fire regimes in tropical rain forests increase the occurrence of fire weather, contributing to a fire-feedback. In Amazonia, for example, aerosols in smoke from deforestation fires can inhibit rain-cloud formation, thereby lengthening the fire season by 15–30 days (Bevan *et al.*, 2009), thus creating a powerful feedback. Smoke plumes also enhance the power and frequency of positive cloud-to-ground lightning, the lightning type most strongly associated with wildfire ignitions. Because of long-distance smoke transport, these effects can last for months and their influence extends well beyond the region where the fires occur. For example, smoke from fires in southern Mexico has been shown to increase the number of positive cloud-to-ground lightning strikes as far away as Ontario, Canada (Lyons *et al.*, 1998).

### Fire in intermediate primary productivity environments

In the tropical savanna biome, frequent fires are common, given the seasonal occurrence of fire weather and a high incidence of lightning. Here, humans influence fire regimes but to a much lower degree than in tropical rain forests (Archibald *et al.*, 2009). For example, in Kruger National Park, South Africa, an analysis of a 50-year record showed that strongly contrasting styles of fire management had no substantial effect on area burnt or fire-free interval, but human ignitions strongly influenced the seasonal and spatial patterns of fires and thus, possibly, reduced fire intensity (van Wilgen et al., 2004). In southern Africa, Archibald *et al.* (2009) showed that burn area decreased with increased grazing, road density and human population density.

### Fire in low primary productivity environments

Arid biome fires typically follow periods of above-average rainfall that produce sufficient biomass to carry fire (Bradstock, 2010). However, humans can change these systems by overgrazing and/or introducing flammable plant species that change the spatial and temporal structure of fuels. For example, flammable invasive grasses have made fires more frequent or more severe in the deserts of North America and Australia, thereby transforming large parts of these ecosystems (D'Antonio & Vitousek, 1992; Brooks & Matchett, 2006; Miller *et al.*, 2010).

## Human fire suppression

Suppression of landscape fire by government authorities is increasingly driven by urban settlement in flammable landscapes (Hammer *et al.*, 2007, 2009). This creates a dangerous juxtaposition of flammable vegetation, high densities of humans (who are the source of most ignitions), and associated infrastructure (Radeloff *et al.*, 2005; Syphard *et al.*, 2007). Sophisticated and costly technologies, such as aerial detections of ignitions and the use of aerial bombers to drop fire retardant, have been developed to fight fires on the ‘wildland–urban interface’, in conjunction with the establishment of firebreaks and pre-emptive burning of fuels under moderate weather conditions (Fig. 2). Under extreme weather conditions, these approaches fail in some shrubland and forest types because fuel continuity does not limit the spread of fires (Moritz *et al.*, 2004, 2010). Mechanized fuel treatments are also being carried out across landscapes and can be effective in reducing fire intensity in some dry forests that formerly sustained frequent, low-severity surface fires (Finney *et al.*, 2005). However, mechanical treatments are more controversial in some moist forest types because there is debate about the ecological justification of this method and about its efficacy in reducing large-scale fires (Schoennagel *et al.*, 2009).

Recent catastrophic fires with tremendous losses in terms of property and lives have been experienced in the Mediterranean Basin, Israel, California, South Africa, southern Australia and Russia. These fires have demonstrated that, while fire management agencies on all five continents can reduce the fire hazards in these environments, they cannot completely prevent fires from spreading into urban environments. At the heart of fire management debates on the wildland–urban interface are political and cultural factors (Hammer *et al.*, 2009). The combination of risks to life and property, the development of institutions tasked with controlling fires, and high media interest in the spectacle of agencies fighting uncontrolled fires has resulted in a widespread public perception that all landscape fires are ‘disasters’ that must be controlled, hampering rational debate about options for coexisting with inherently flammable landscapes (Fig. 2). This provides a significant political driver of increasing expenditure on fire management. For example, the US Forest Service is currently spending over US$1 billion year^−1^ fighting increasingly severe fires (González-Cabán, 2008). The use of planning statutes to segregate dwellings from flammable landscapes, to reduce diffuse settlement patterns across wildland–urban interfaces, has been suggested as an option to protect life and property. However, this approach does not enjoy political support and is at odds with the amenity values ex-urbanites place on residing in natural landscapes (Bradshaw, 1988; Buxton *et al.*, 2011). For instance, the compulsory purchase of land by government was one of the few recommendations of the Victoria Bushfire Royal Commission looking into the catastrophic Australian fires of 2009 that was not adopted by the Victorian Government (Teague *et al.*, 2010).

## Humans and global fire activity

Global analyses of past and current fire activity have provided insights into the interaction between climate and anthropogenic burning. Analyses of charcoal in sediment records over the past 21,000 years suggest that fire regimes respond primarily to changes in regional climate and/or climate-induced vegetation changes (Power *et al.*, 2008). Increased fire activity has been shown during periods of rapid climate change (Marlon *et al.*, 2009). In the Last Glacial Maximum, there was less biomass burning than today in most regions of the world, owing to generally colder and drier conditions. Regarding the last 2000 years, however, Marlon *et al.*’s (2008) charcoal analyses show that the dominant effect of climate on fire activity globally has increasingly been influenced by human land use. Charcoal levels decline between ad 1 and 1750, ascribed to the effects of cooling in the late Holocene, and then increase between ad 1750 and 1870, attributed to forest clearance in the Americas, Europe and Australia. Between ad 1870 and 1950, decreasing charcoal levels are explained by land-use changes and practices that have reduced fire prevalence, including forest clearance, grazing, and fire suppression policies. Nonetheless, such global syntheses that rely on charcoal records are unable to capture regional heterogeneity. Also missed by this type of palaeoecological synthesis are abrupt changes in fire regimes associated with the first arrival of humans into infrequently burnt biomes (McWethy *et al.*, 2010). Fire and climate histories for the past 1000 years derived from documentary sources, fire scars on tree rings, and charcoal in lake sediments from western North America indicate that climate has been the predominant control of inter-annual to decadal variability in fire regimes in conifer forests (Kitzberger *et al.*, 2007). However, in many biomes there is insufficient data to allow us to disentangle anthropogenic and ‘natural’ fire effects and their interactions. For example, historically some of the most densely populated landscapes were in the semi-arid lowlands of the southern Pacific coast of North America, and these environments are often devoid of the charcoal records necessary for studying Holocene fire activity (Keeley, 2002).

Over the last 300 years, global human impacts have intensified. Ellis *et al.* (2010) suggest that only 5% of the ice-free land surface had been substantially modified by humans for agriculture and settlement just prior to the start of the Industrial Revolution, but by ad 2000 this had increased to 55%. This global landscape transformation was associated with widespread clearing and burning of forests to create farmland and with the combustion of large quantities of fossil fuels. Combined, these sources of combustion overturned the old relationships that had defined anthropogenic burning for thousands of years. Indeed, the concept of an ‘Anthropocene’, a geological time interval defined by anthropogenic impacts on the Earth system (Steffen *et al.*, 2011; Zalasiewicz *et al.*, 2011), took as its point of origin the late 18th century, when industrial combustion became prominent in many economies (Crutzen, 2002). Beyond consideration of the impacts on atmospheric chemistry and climate, however, the cascade of ecological consequences of anthropogenic burning since the Industrial Revolution has not been tracked systematically. Indeed, this historical process has not been recognized as a prominent question for fire ecology. Among the few attempts, Pyne (2001) developed the concept of the ‘pyric transition’ to highlight humanity's shift in the type and scale of fire practices accompanying industrialization. The pyric transition concept alludes to the well-known ‘demographic transition’ that describes changes to human population as a consequence of industrialization (Kirk, 1996). In this case, pyric transition refers to landscape fire activity that irrupts from background levels, and then collapses below that level as humans use fossil fuels and the internal combustion engine to replace or suppress landscape burning. Because fire can be as ecologically powerful when removed as when applied, the pyric transition has had substantial consequences for fire management. Globally, it is very difficult to disaggregate the influence of humans on fire regimes (Krawchuk *et al.*, 2009; Lauk & Erb, 2009), so the pyric transition concept is yet to be subjected to a quantitative analysis.

## Model of global pyric phases

**Figure 3:**
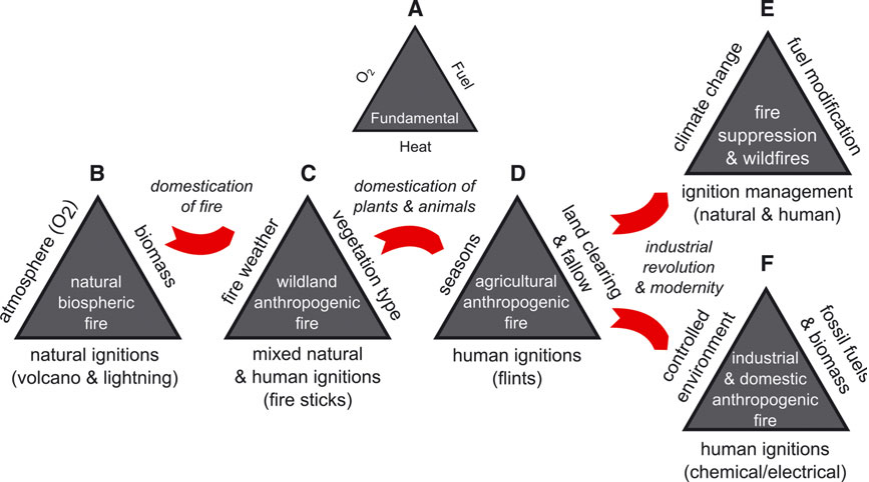
Schematic representation of the model of global pyric phases. The model is based on the classical fire triangle concept, which represents fire as a physiochemical process made up of three vital ingredients: oxygen, heat and fuel (A). With the evolution of terrestrial vegetation, fire was able to become a biospheric phenomenon, given lightning and volcanic ignitions and sufficient oxygen in the atmosphere. Fire activity varied in response to oxygen levels and vegetation types (B). Prehistoric humans domesticated fire, leading to modification of vegetation, by setting fires under suitable weather conditions. The motive for burning varied and included game and habitat management. These prehistoric traditions remain important in many contemporary wildlands, albeit in modified forms (C). Fire is an important tool for clearing land to establish fields and is incorporated into many agricultural systems to burn dead biomass in specific seasons to prepare fields for cultivation, remove post-harvest residues and stimulate pasture growth (D). Industrialization has influenced landscape fire activity by changing ignition patterns, enabling the development of suppression technologies and causing climate change via greenhouse gas pollution (E). Fossil fuels increasingly replaced biomass as an energy source following industrialization (F). All phases remain on Earth, although comparative studies remain rudimentary.

Humans are the keystone species for fire on Earth, and there is a continuity between biomass burning by hunter–gatherers and then agriculturalists and later fossil fuel consumption following the Industrial Revolution. We suggest that studying the transition from one style of fire management (pyric phase) to another is a key to understanding the dynamics of human and fire interactions, and the consequences that have cascaded through Earth systems and human societies (Fig. 3). For instance, the Aboriginal tradition of frequent patchy fires in northern Australia savannas, which created a fine-scale mosaic of burnt and unburnt areas (Bowman *et al.*, 2004), has been replaced with frequent, very large fires in many areas under European management (Russell-Smith *et al.*, 2007), with an increase in the abundance of flammable grass fuels (Bowman *et al.*, 2007). In some pine-dominant, semi-arid forests of western USA, fire regimes in the prehistoric period were characterized by frequent (< 20-year interval) and extensive surface fires ignited by spatially and temporally varying combinations of lightning and people. European colonization led to a switch from surface fire regimes to infrequent and severe crown fires (Veblen *et al.*, 2000), because of changed fuels that resulted from livestock grazing and fire suppression, the latter using a variety of technologies supported by the State. Conversely, in Mediterranean Basin landscapes the migration of rural populations to metropolitan centres over the last half-century has led to land abandonment and subsequent reduction in livestock grazing. Pastures and grazing areas reverted to shrubland, and this has led to coalescence of fuel continuity, contributing to an increase in large fires (Moreira *et al.*, 2001).

Because all pyric phases in our model (Fig. 3) are still apparent on Earth they remain visible across current landscapes as chronosequences and are evident in historical time series such as documentary records and palaeoreconstructions. For example, Korontzi *et al.* (2006) used Moderate Resolution Imaging Spectroradiometer (MODIS) satellite detections of active fires to describe global patterns of agricultural fire use between ad 2001 and 2003, demonstrating that about one-third of all agricultural fires occurred within the Russian federation. Le Page *et al.* (2010) have highlighted the global imprint of humans on fire activity by analysing the discrepancies between historical and current fire activity and that expected from lightning ignitions, fuel production and fire weather.

**Figure 4:**
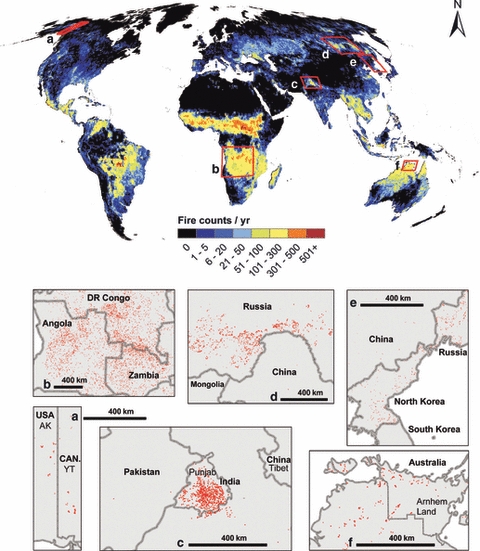
Global distribution of fires generated by human and natural causes, represented as active fire counts per year recorded with the MODIS sensor (Terra) between 2001 and 2007 (Giglio *et al.*, 2006). Panels illustrate fire activity on selected cloud-free days at various locations spanning political boundaries where differences in fire management policy and cultural practices may (c, d, e) or may not (a, b, f) affect fire activity. Images were provided by the MODIS Rapid Response Team at NASA GSFC. http://lancedev.eosdis.nasa.gov/imagery/rapid-response/.

Disentangling the influence of human land uses on background fire regimes is a major research challenge, requiring detailed comparative palaeoecological analyses of the effects of past fire activity by contrasting human land use regimes within similar biomes (Whitlock *et al.*, 2010). Likewise, there are sharp contrasts in fire activity within the same biome owing to different styles of land management between adjacent regions. Such geographic patterns provide opportunities to examine ‘natural experiments’ to discern social and political drivers and ecological and economic consequences (Fig. 4). Such comparative studies require the development of a lexicon and methodology to describe fire regimes, their biophysical consequences, their political and cultural contexts, and economic costs and benefits (Chuvieco *et al.*, 2008).

## Future global fire regimes

Recent warming trends have been associated with an increase in fire activity in some regions (Hennessy *et al.*, 2005; Westerling *et al.*, 2006; Flannigan *et al.*, 2009; Spracklen *et al.*, 2009), but whether or not this is a manifestation of global climate change remains controversial. Thus, whether extreme fire events, such as Australia's Black Saturday bush fires of February 2009 (Cameron *et al.*, 2009), can be attributed to climate change remains unknown. A positive feedback between climate change and biomass burning is plausible given the direct impact of fire on biogeochemical cycles, particularly carbon fluxes (Bowman *et al.*, 2009). For example, increased atmospheric CO_2_ may change the mass or combustibility of different fuel types, owing to altered growth rates and competitive abilities, influencing fire regimes and vegetation patterns (Ziska *et al.*, 2005; Wigley *et al.*, 2010). Nonetheless, it is widely assumed that, at the global scale, long-term (i.e. decadal to longer) effects of fire on carbon fluxes are largely cancelled by vegetation regrowth following fire (Solomon, 2007). However, with rapidly changing climates and large social and industrial changes, this assumption may be increasingly (and dangerously) wrong (Bowman *et al.*, 2009). The vast areas of western Russia that were burnt in the summer of ad 2010 provide an example of how anthropogenic global climate change (ACIA, 2004) and socio-political circumstances leading to dysfunctional fire management can alter fire regimes at regional to global scales (Achard *et al.*, 2008).

Fire is a natural disturbance in the boreal biome (Kurz & Apps, 1993) that was apparently controlled by weather and fuel moisture in pre-modern times (Chapin *et al.*, 2006). However, anthropogenic climate change may increase the number of days suitable for burning (Kasischke, 2000), with a corresponding increase in the frequency, intensity and areal extent of fires (Kurz & Apps, 1999; Kasischke & Turetsky, 2006; Flannigan *et al.*, 2009). Increased burning in boreal forests could potentially provide a positive feedback on global warming through the release of greenhouse gases and particulates (Kasischke, 2000; Randerson *et al.*, 2006) and the mobilization of vast quantities of carbon currently stored in frozen soils (Zimov *et al.*, 2006). Alternatively, fire could slow regional warming through the alteration of ecosystem energy balances by changing albedo; for example, reduced tree cover exposes proportionally more snow, thus increasing reflectance (Randerson *et al.*, 2006; Euskirchen *et al.*, 2009). The resolution of this question is of global significance, given that boreal forests sequester 88 Pg C (25% of the global vegetation C pool) (Dixon *et al.*, 1994) and the soils and sediments of arctic and boreal regions store over 1672 Pg C – a pool more than twice the size of the atmospheric C pool. The extensive fires in western Russia highlight how policy and human land use changes can exacerbate the warming trends observed in boreal regions. These wildfires were clearly associated with extraordinarily warm temperatures that repeatedly exceeded previous daily maximums in thermometer records for 130+ years. Humans are suspected as an ignition source for many of the fire complexes, for reasons ranging from carelessness to economic advantage (Mollicone *et al.*, 2006; Sofronova *et al.*, 2010). For example, logging of burnt forests is permitted by local and regional authorities, thus proving an incentive to set fires in protected forests.

Managing climate, fire, carbon and economic feedbacks in a period of rapid global environmental change necessarily demands consideration of a human dimension to fire regimes across different biomes. At a global scale, uncontrolled fire activity could potentially compound climate change through fire-related greenhouse gas emissions and associated feedbacks. At a local to regional scale, humans must learn to use fire while neither degrading biodiversity and ecosystem services nor threatening human health and wellbeing, and approaches will necessarily vary amongst biomes. In tropical rain forests, nearly all fires are of anthropogenic origin, so eliminating wildfire from this system would have substantial greenhouse gas emission benefits: tropical rain forest wildfires release between 7.5 and 70 Mg ha^−1^, depending on previous fires, land use history and forest region (Cochrane *et al.*, 1999; Balch *et al.*, 2008), and tropical peat fire events are more severe, possibly exceeding 300 Mg C ha^−1^ (Page *et al.*, 2002). In flammable biomes, such as tropical savannas and seasonally dry forests, there is often a conflict between fire regimes that support biodiversity and those regimes designed to reduce fire risk to humans and their infrastructure or that support livelihoods such as pastoralism and forestry (Laris, 2002). Global climate change, with associated historically anomalous fire weather, will exacerbate these tensions, as will proposals to change fire regimes in order to reduce greenhouse gas emissions from fires and increase terrestrial carbon stocks (Williams *et al.*, 2004; Laris & Wardell, 2006). Clearly, the management of flammable environments must change with increasing extreme fire weather, yet there remains uncertainty as to the most appropriate and sustainable strategies. Improved knowledge of the following aspects of fire is needed: (1) a better understanding of past fire regimes, (2) how humans currently influence the regional variation in contemporary burning practices, (3) the underlying planning regulations and economic costs and benefits of different types of fire use, (4) social and political responses to risk of fire, and (5) the economic and ecological costs and benefits of fire.

Global modelling, for a range of plausible scenarios, is a critical step in exploring the interactions of greenhouse gas emissions, climate change and human fire usage. The human dimension of fire management has been poorly characterized in global models of fire activity (Lavorel *et al.*, 2007), although more effort is being directed in this area. Several modelling studies have shown that increasing global temperatures could lead to increases in fire occurrence in some areas of the globe, but to decreases in others (Scholze *et al.*, 2006; Krawchuk *et al.*, 2009; Pechony & Shindell, 2010). Pechony & Shindell (2010) combined global fire and climate modelling to describe changes in fire activity from the pre-industrial period into plausible future climates by assuming that both human ignitions and suppression increased with human population density. While they noted high levels of uncertainty and a paucity of global data on past fire activity, their modelling revealed a switch from moisture-limited fire activity in the pre-industrial period, when human populations were low, to anthropogenic-driven ignition regimes as the Industrial Revolution proceeded in step with human population growth. They predict that, in the 21st century, fire activity may become controlled by increased temperature, overwhelming both human ignitions and suppression efforts. More refined modelling could identify regions where fire and ecosystem management strategies are most likely to have the greatest benefit in reducing greenhouse gas pollution. For example, intentional modification of fire regimes to reduce fire intensities, thereby decreasing greenhouse gas emissions and increasing carbon storage in some landscapes, has been proposed as a strategy to mitigate climate change (Williams *et al.*, 2004; Hurteau *et al.*, 2008), although the global benefit of these interventions remains unknown. Global modelling could also aid in predicting where regional transitions from fire-sensitive to fire-promoting vegetation, such as the grass–fire cycle (D'Antonio & Vitousek, 1992), are most likely to occur.

## Conclusions

The ancient human–fire relationship blurs the distinction between natural and anthropogenic fire regimes. Many human cultures appear to have achieved an enduring coexistence with fire, often by domesticating it, as has been done with many plants and animals. In some situations, the development of this coexistence is known to have had substantial ecological effects on the environment. However, the transition from local-scale fire use to the global industrialization that has triggered climate change requires that we turn our attention to the effects of altered fire regimes on the Earth system. This requires better understanding of the diversity of human fire use, especially possible positive and negative feedbacks across a range of scales. This project demands integrative, multidisciplinary perspectives on landscape fire, its ecological effects and relationships with human societies, spanning geographic scales from the local to the global, whilst retaining an ecological and evolutionary frame of reference. Comparative studies of past and current human influences on fire regimes amongst biomes are required to identify excursions from the historical range of variability, a key step in identifying locally sustainable and unsustainable human–fire relationships. An understanding of different cultural traditions and political (local to geopolitical) influences in the management of fire is essential for evaluating the costs and benefits of contrasting fire regimes within individual landscapes and biomes. For example, Indonesia's decision in the 1980s and 1990s to promote the drainage of carbon-rich peatlands has led directly to catastrophic carbon releases, conservatively estimated at 2–3 Gt (Page *et al.*, 2009). Combustion of these peatlands is strongly controlled by drought, which may become more severe with climate change (van der Werf *et al.*, 2008). Brazil's recent policy changes have contributed to reducing Amazonian deforestation rates dramatically by over 70% (Artaxo, 2010), but climate models predict a high vulnerability of eastern Amazonia to climate change. Collectively, these insights will enable better representation of the diversity of anthropogenic fire regimes in dynamic global vegetation models, which are crucial to understanding the carbon cycle and identifying strategies to manage fire so as to reduce emissions and increase carbon storage. Such an integrated research programme is essential to enable humanity to coexist sustainably with our inherently flammable planet.

## Biosketch

The paper is a product of a National Center for Ecological Analysis and Synthesis Pyrogeography working group organized by D.M.J.S.B. and J.K.B.

Author contributions: D.M.J.S.B. led the writing with contributions from all authors. The figures and tables were developed by D.M.J.S.B., J.K.B., W.J.B., C.A.K., M.A.K., S.J.P., A.C.S. and T.S.
